# Integrating Physiotherapy for Enhancing Functional Recovery in Glioblastoma Multiforme: A Case Report

**DOI:** 10.7759/cureus.57199

**Published:** 2024-03-29

**Authors:** Ghanishtha C Burile, Raghumahanti Raghuveer, Vaibhav Chandankhede, Shrushti Jachak, Neha Arya

**Affiliations:** 1 Neurophysiotherapy, Ravi Nair Physiotherapy College, Datta Meghe Institute of Higher Education and Research, Wardha, IND; 2 Otolaryngology-Head and Neck Surgery, Indira Gandhi Government Medical College and Hospital, Nagpur, IND

**Keywords:** quality of life, palliative care, rehabilitation, neurosurgery, grade iv glioma, tumor suppressor genes, gliomagenesis, headache, glioblastoma multiforme

## Abstract

Glioblastoma is the most prevalent primary brain tumor. Because glioblastomas are very vascular, they may worsen the disease's neurologic symptoms by causing vasogenic brain edema and mass effects with a wide range of other symptoms. In this case report, a 42-year-old male complaining of severe headache, generalized weakness, and forgetfulness was brought to a territory care hospital, where a detailed neurological examination and investigations with magnetic resonance imaging (MRI) revealed a grade IV (high-grade) glioma at the right frontotemporal and capsuloganglionic regions of the brain, and was suggested for surgery. Postoperatively, the patient was referred for chemotherapy, but due to severe weakness, fatigue, and motor deficits, he was referred for physiotherapy. Follow-up was conducted to monitor the patient's progression using various outcome measures. These measures included the Functional Independence Measure (FIM), the Intensive Care Unit (ICU) Mobility Scale, the Glasgow Coma Scale (GCS), the modified Rankin Scale (mRS), and the Karnofsky Performance Status (KPS) Scale. Significant improvement was observed in the patient's symptoms, as tracked by these outcome measures. Therefore, it is important that a tailored rehabilitation protocol of six weeks was planned, focusing on palliative care and some symptoms of weakness, reduced strength, tone, and breathlessness to prevent secondary complications like deep vein thrombosis, irritability, anxiety, forgetfulness, decreased balance, and coordination in sitting. Since the prognosis of grade IV glioblastoma is poor, the goal-oriented rehabilitation program will help improve the palliative status and the overall quality of life of the patient.

## Introduction

Most malignant brain tumors in humans, glioblastoma multiforme (GBM), can arise de novo (primary glioblastoma) or due to anaplastic astrocytoma (secondary glioblastoma), called grade IV astrocytoma. It grows rapidly and is aggressive in nature [1]. In India, the prevalence of GBM was between 23,000 and 49,000 in 2016. GBM is more common in men than in women, with a ratio of 1.6:1 [2]. In some research, reports link occupation, environmental carcinogens, and food (N-nitroso compounds) to a higher risk of developing gliomas. Nevertheless, therapeutic X-irradiation is the only environmental component that is unquestionably linked to an increased risk of brain tumors, including gliomas [3]. The most typical early sign of brain tumors is a headache. It may exhibit migrainous or tension-type characteristics, which get worse by lying flat, and as a result, they are most apparent while waking up. Clinical features of GBM are headache seizures, dysphagia, aphasia, lethargy, dyspnoea, motor weakness, disorientation, sleepiness, and depression [4]. According to the World Health Organization (WHO), grades II and III are categorized as low-grade gliomas (LGG) under this classification. Many of these tumors have the potential to progress to grade IV GBM, which has a worse prognosis if not treated as early as possible and is highly malignant [5].

The multistep process of gliomagenesis, or the formation and development of gliomas, involves genetic changes in normal cells that result in the production of malignant derivatives. Gradually, cells undergo genetic and epigenetic modifications that lead to the inhibition of tumor suppressor genes (TSG) and the activation of proto-oncogenes [6]. Pilocytic astrocytoma of the posterior fossa is a highly malignant (grade IV) glioma that does not spread and can be surgically cured. In contrast, glioblastoma has a median survival of less than 12 months and is more likely to invade the entire brain [7-9]. Within 30 days following tumor excision, the following problems are common which include stroke, myocardial infarction, infection, mortality, and the necessity for revision surgery [10,11]. Glioma patients have a wide range of palliative care requirements, including requirements for physical, psychological, social, and spiritual support. Rehabilitation plays a very crucial role in the prevention of postoperative complications after any neurosurgery, like deep vein thrombosis and pressure sores due to prolonged bed rest, reduces the length of hospital stay, decreases time on ventilation, improves muscle strength, and increases independence in activities of daily living to improve functional capacity and quality of life [12,13].

## Case presentation

A 42-year-old male patient was brought to a tertiary care hospital by relatives with complaints of headache for 15 days and generalized weakness for 10 days. The patient was referred for a magnetic resonance imaging (MRI) of the brain and was diagnosed with high-grade glioma at the right frontotemporal and capsuloganglionic regions. Then, he was admitted to the hospital, and a glioma excision with right frontotemporal craniotomy was done on 7/02/24. After surgery, he was shifted to the neurology intensive care unit (ICU), where he was on a mechanical ventilator. He does not have any comorbidity. He was administered an injection of ceftriaxone 1 gm, an injection of metronidazole 40 mg, and an injection of mannitol 100 ml medications. The patient was referred for physiotherapy to reduce the postoperative complications and for palliative care.

Clinical evaluation

Patient informed consent was taken before evaluation and treatment. The assessment was taken on postoperative day 4 in the ICU. The Glasgow Coma Scale (GCS) score was E3V1M2. After improving consciousness, the Mini-Mental State Examination (MMSE) score was taken which was 2 (severe degree of impairment). A detailed neurological examination was performed. No sensory abnormalities were seen. Muscle tone examination revealed hypotonia in the left upper and lower limbs according to the tone grading scale. After improving consciousness and muscle tone, manual muscle testing was also done. Deep tendon reflexes for the left side were found to be grade 1+ (diminished), and for the right side, they were grade 2+ (normal). Babinski's sign was positive.

Investigations

**Figure 1:**
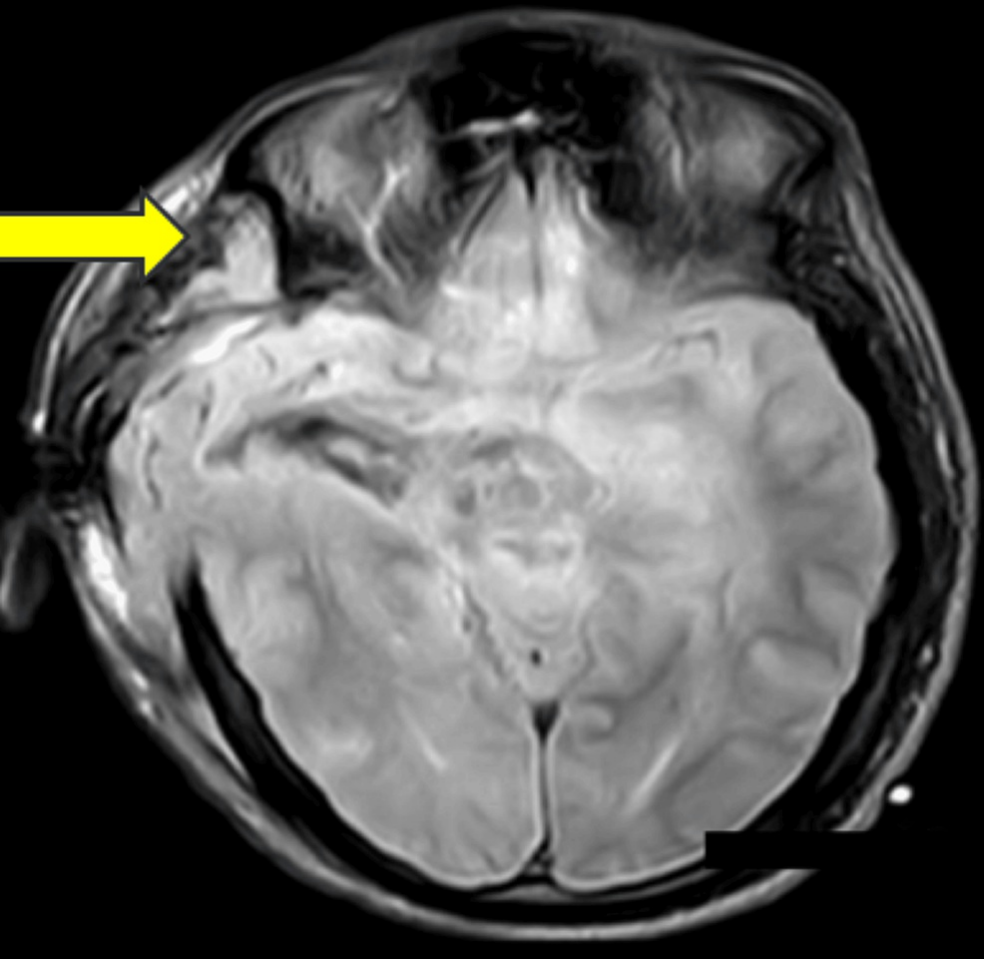
MRI of the brain revealed high-grade glioblastoma multiforme/astrocytoma indicated with a yellow arrow. MRI: magnetic resonance imaging

MRI of the brain was done, which revealed there was a defined lesion with solid cystic components involving the right basal ganglia, corona radiata, and centrum semiovale. High-grade GBM/astrocytoma was prominently visible in Figure 1.

Physiotherapy intervention protocol

A detailed physiotherapy management protocol was tailored according to the severity of symptoms. The rehabilitation program was planned for six weeks based on the principles of palliative care, focusing on the prevention of postoperative complications and improving functional capacity and quality of life as mentioned in Table 1.

**Table 1:** Rehabilitation protocol ROM: range of motion; DVT: deep vein thrombosis; PNF: proprioceptive neuromuscular facilitation

Problem list	Goals	Interventions	Repetitions
Impaired consciousness	To improve the level of consciousness, enhance sensory awareness, and assist the patient in being more aware of their own body	Multimodal stimulation: the patient can be stimulated with a variety of visual, aural, and olfactory stimuli, including applying ice, utilizing tactile materials, playing music, or introducing familiar fragrances	Once or twice a day
To prevent secondary complications like muscle tightness and ROM	To maintain joint integrity and ROM	Passive ROM exercises of bilateral upper and lower limbs (10 repetitions × 1 set). Then, it can be progressed to active assisted ROM exercises	10 repetitions × 2 sets
Reduced muscle tone	To initiate movement and improve muscle tone	PNF D1 flexion-extension (rhythmic initiation) for upper and lower limbs; Rood's facilitatory approach; icing (2-3 times/day)	10 repetitions × 2 sets
The patient feels breathlessness while performing the exercise	To reduce fatigue and symptoms of breathlessness	Diaphragmatic breathing and activity pacing may enhance physical activity and reduce fatigue levels [14]	10 repetitions × 3 sets
Irritability and anxiety	To reduce the symptoms of anxiety	Benson relaxation technique: sit comfortably and quietly by closing your eyes, relax the muscles of your whole body, and breathe naturally and slowly [15]	For 20 minutes, follow this technique everyday with an empty stomach
Prone to complications of DVT and bed sore	To reduce the risk of complications of DVT and bed sore	Compression stockings, positioning strategy, and pillow positioning under the ankle joint	20 minutes x 4 times/day
Reduces functional activity	To improve functional activity	Bed mobility exercises: bedside rolling, transfer training from sitting, and weight shifts to improve static balance with maximal assistance	2-3 times a day

Outcome measures

Pre- and post-rehabilitation outcome measures were taken to note the progression of the condition (follow-up) as mentioned in Table 2. This was a case of grade IV glioblastoma in which the prognosis was poor, but with the help of a multidisciplinary team and a tailored rehabilitation protocol that was followed for about six weeks, there was significant improvement seen in the symptoms of the patient. Post-rehabilitation after six weeks, the patient succumbed due to cardiac arrest. As the prognosis of the patient was poor, via physiotherapy, we tried to reduce the patient's symptoms and improve quality of life.

**Table 2:** Outcome measures FIM: Functional Independence Measure; ICU: Intensive Care Unit; GCS: Glasgow Coma Scale; KPS: Karnofsky Performance Status; mRS: modified Rankin Scale

Outcome measures	Pre-rehabilitation	Post-rehabilitation
FIM	Level 1 (total assistance)	Level 2 (maximal assistance)
ICU Mobility Scale	0 (nothing, lying in bed)	1 (sitting in bed, exercise in bed)
GCS	6	11
mRS	5 (severe disability, bedridden, incontinent, and requires nursing care and attention)	4 (moderate-severe disability, unable to walk without assistance, and unable to attend to own bodily needs without assistance)
KPS	20 (very ill, urgently requiring admission, requires supportive measures of treatment)	50 (requires help often, requires frequent medical care)

## Discussion

Glioblastoma is a malignant, fast-growing astrocytoma. Intrinsic brain tumors such as glioblastomas are believed to develop from neuroglial stem or progenitor cells. Tumors of the isocitrate dehydrogenase (IDH) wild type comprise more than 90% of glioblastomas [16]. Malouff et al. researched that Boron neutron capture therapy has a high linear energy transfer and immunotherapy keeps lymphocytes engaged; these two treatments can be used in tandem to maximize the synergy between immune activation [17,18]. Liao and coauthors' research on nanocarriers has attracted much attention in the research of GBM owing to their benefits in self-assembly, biosafety, release controllability, and blood-brain barrier penetrability, making them promising candidates for GBM treatment [19]. In some research, it was found that there is a comparatively low prevalence of proactive advance care planning and effective palliative care services among GBM patients, even though these elements are essential to providing high-quality care for these patients and their caregivers [20]. The case presented here highlights the challenges faced by a 42-year-old male diagnosed with grade IV GBM, which is an aggressive form of brain tumor. The patient initially presented with severe headache, generalized weakness, and forgetfulness, which led to the discovery of the tumor in the right frontotemporal and capsuloganglionic regions. Following surgery for tumor excision, the patient experienced complications requiring intensive care, including mechanical ventilation. Physiotherapy interventions used in the above case report are diaphragmatic breathing exercises to improve the functioning of the diaphragm as the patient was on a ventilator, passive range of motion exercises, proprioceptive neuromuscular facilitation (PNF) techniques to improve muscle tone and quality of movement, prevention of postoperative complications like bed sores, deep vein thrombosis positioning, bed mobility exercises, and compression stocking, and all these physiotherapy interventions played an important role in improving the patient's quality of life.

The significance of the physiotherapy interventions was assessed through various outcome measures, including the Functional Independence Measure (FIM), ICU Mobility Scale, GCS, modified Rankin Scale (mRS), and Karnofsky Performance Status (KPS) Scale. Postoperatively, improvements were observed in both functional independence and cognitive function, albeit with varying degrees of progress. The importance of physiotherapy and palliative care in the management and recovery of patients with GBM cannot be overstated. While the prognosis for grade IV glioblastoma remains poor, comprehensive rehabilitation programs can significantly improve the quality of life and functional outcomes of the patient. By addressing both physical and cognitive impairments, physiotherapy plays a crucial role in optimizing recovery and maximizing the potential for meaningful improvements in patients' lives.

## Conclusions

The above case underscores the critical role of physiotherapy in the multidisciplinary management of grade IV GBM. By addressing the severity of symptoms through tailored rehabilitation programs, slight improvements in consciousness, functional independence, and cognitive function can be achieved postoperatively. Despite the poor prognosis associated with GBM, a comprehensive approach that includes physiotherapy interventions holds promise in enhancing the quality of life and overall well-being of affected individuals. Further research should be focused on novel treatment modalities focusing on increasing life expectancy through advanced rehabilitation. Overall, collaboration between neurosurgery, rehabilitation medicine, and other disciplines is essential to maximize treatment efficacy and enhance patients' quality of life in the face of this devastating disease.
